# Diagnostic Labeling Patterns of Malnutrition and Undernutrition in Japan: A Nationwide Patient Estimation Database Study

**DOI:** 10.3390/nu18091337

**Published:** 2026-04-23

**Authors:** Mari Maese, Shingo Kondo, Takeru Saito, Yuko Okamoto, Hiroki Iwata, Noriko Kobayashi, Katsunori Yamaura

**Affiliations:** 1 Division of Social Pharmacy, Center for Social Pharmacy and Pharmaceutical Care Sciences, Faculty of Pharmacy, Keio University, Tokyo 105-8512, Japan; mari.m-1128@keio.jp (M.M.); takeru.saito.021006@keio.jp (T.S.); okamoto-yk@keio.jp (Y.O.); iwahiro@keio.jp (H.I.); kobayashi-nr@keio.jp (N.K.); yamaura-kt@keio.jp (K.Y.); 2 Keio University Community Pharmacy, Tokyo 105-8512, Japan

**Keywords:** sex difference, International Statistical Classification of Diseases and Related Health Problems, global leadership initiative on malnutrition

## Abstract

**Background/Objectives**: Malnutrition and undernutrition are critical health concerns associated with increased mortality and costs. Although these are distinct clinical concepts, they are often used interchangeably in clinical practice and are inconsistent with the diagnostic frameworks. This diagnostic ambiguity may obscure true patient profiles. This study aimed to clarify the real-world diagnostic patterns of malnutrition and undernutrition and identify associated drug prescription trends using a patient estimation database in Japan. **Methods**: We analyzed the AHI partners database of 2024. Patients were identified using the International Statistical Classification of Diseases and Related Health Problems, 10th Revision, code E46. Sex differences were analyzed and stratified according to age group: older adults (≥65 years) and younger adults (15–39 years). Odds ratios (ORs) were used to identify associated drugs. **Results**: Of 96,673,453 patients, 216,652 were diagnosed with malnutrition and 77,100 with undernutrition. In both categories, older adults accounted for more than half of the patients. Notably, distinct diagnostic labeling patterns were observed by sex. Malnutrition predominated in women (58.8%), whereas undernutrition was more prevalent in men (70.6%). This male predominance of undernutrition was reversed in younger adults, where women showed higher proportions in both categories. Prescription analysis identified 31 drugs frequently prescribed to the study population. Enteral elemental formulas had the highest OR (89.7). Some psychotropic drugs were frequently prescribed to women. **Conclusions**: Diagnostic patterns varied by sex and age, potentially reflecting distinct practices in diagnostic labeling. These findings highlight the need for standardized frameworks to ensure consistent assessments and effective nutritional interventions.

## 1. Introduction

Two critical terms encompass the concept of nutrition: malnutrition and undernutrition. Malnutrition is a substantial concern that is associated with poor patient outcomes. According to the World Health Organization (WHO), malnutrition is defined as “deficiencies, excesses, or imbalances in a person’s intake of energy and/or nutrients, or impaired utilization of nutrients” [1], encompassing undernutrition and overnutrition [2]. Among older adults, adverse outcomes related to malnutrition are often more complex and severe when associated with undernutrition than when associated with being overweight or obese [3]. Undernutrition refers to a condition in which essential nutrients, such as proteins and energy, are insufficient to maintain health [2]. Undernourished individuals have a 1.6- to 4.4-fold higher risk of mortality [4] than well-nourished individuals. Furthermore, undernutrition is associated with more than a 2-fold increase in healthcare costs within 6 months after diagnosis (£1753 vs. £750) [5], highlighting the substantial burden on individuals and healthcare systems [6]. Undernutrition is a pervasive problem in Japan. According to the 2023 National Health and Nutrition Survey, the prevalence of undernutrition tendency, defined as a body mass index (BMI) ≤ 20 kg/m^2^, among individuals aged ≥65 years, considered older adults in Japan, was approximately 12% in men and 22% in women [7]. However, this vulnerability is not exclusive to older adults. Recent evidence highlights the critical risk of malnutrition among young Japanese women who pursue an extremely low body weight, which corresponds to a BMI of 18 kg/m^2^ [8]. This underscores the importance of addressing undernutrition as a life-long issue rooted in physiological and sociocultural factors. Consequently, undernutrition must be recognized as a significant public health concern throughout life rather than an issue confined to older populations. Women are at an increased risk of undernutrition [9,10,11]. However, the interpretation of these sex-based differences remains unclear. The clinical presentation of malnutrition varies according to sex. Women are more likely to present with low body weight or BMI, whereas men are more likely to exhibit loss of muscle mass, a higher burden of comorbidities, and inflammation-related features [12]. Such differences in clinical presentation may influence the recognition, diagnosis, and recording of undernutrition in routine clinical practices. Consequently, sex differences observed in administrative or claims databases may primarily reflect differences in diagnostic labeling rather than true differences in the biological disease burden. This issue is complicated by inconsistencies in the diagnostic criteria and disease classification systems. In the International Statistical Classification of Diseases and Related Health Problems, 10th Revision (ICD-10), undernutrition is classified under code E46 (unspecified protein–energy malnutrition), which includes the broader term malnutrition. In clinical practice, malnutrition and undernutrition are often used interchangeably [3,13,14]. Although the Global Leadership Initiative on Malnutrition (GLIM) criteria [15] are widely recommended, they are designed to only identify undernutrition, despite using the term “malnutrition.” This discrepancy may lead to medical records in which undernutrition and overnutrition are inadequately distinguished in clinical coding. Consequently, in database studies that rely on ICD-10 codes to identify study populations, the true characteristics of patients with undernutrition may be obscured, potentially influencing the analytical results and their interpretations.

Women have a higher risk of developing adverse drug reactions than men owing to factors such as differences in cytochrome P450 activity [16]. We previously reported that information on sex differences in pharmacokinetics and pharmacodynamics in prescription drug package inserts in Japan included only 76 ingredients (4.5%) out of 1679 [17]. An insurance database analysis using ICD-10 codes revealed that women were more likely to experience hypokalemia among patients taking diuretics associated with fluid reduction [18]. Based on these reports, we hypothesized that sex-based differences in diagnostic labeling patterns exist between “malnutrition” and “undernutrition.” This study aimed to clarify the real-world diagnostic patterns of malnutrition and undernutrition using a patient estimation database and to examine how these classification issues may affect sex-specific analyses. Additionally, this study aimed to identify drugs frequently prescribed to patient groups diagnosed with malnutrition or undernutrition.

## 2. Materials and Methods

### 2.1. Study Design

This retrospective cross-sectional analysis used a nationwide patient estimation database in Japan. This study was conducted in accordance with the ethical principles of the Declaration of Helsinki. Anonymized open data without personally identifiable information were used; therefore, ethical approval was not required.

### 2.2. Data Source

Data were obtained from the AHI partners database, an online search system (AHI Partners, Inc., Tokyo, Japan). The AHI partners database is a nationwide patient estimation database constructed based on the Survey of Medical Care Benefit Status published by the Ministry of Health, Labour and Welfare (MHLW) of Japan. This survey covers approximately 99% of the Japanese population, across multiple public health insurance systems. To estimate the nationwide patient numbers, the database adjusted raw claims data using expansion coefficients derived from MHLW reports. These coefficients were calculated by considering factors such as prefecture, age group, medical fee category, and disease-specific age distribution. Through this calibration process, the database provides data comparable in scope and representativeness to that of the National Database of Japan. The database provides information on patient demographics, clinical diagnoses coded according to ICD-10, clinical departments, and prescribed drugs in routine clinical practice, excluding body weight, vital signs, and laboratory test results.

### 2.3. Study Population and Data Extraction

Data were extracted from patients diagnosed with malnutrition-related conditions between 1 January and 31 December 2024. Patients were identified using the ICD-10 code E46 (unspecified protein–energy malnutrition), which included diagnoses of malnutrition (ID 20083992), undernutrition (ID 20105080), protein deficiency disorders (ID 20070284), and malnutrition-related cataracts (ID 20054844). The extracted data included the estimated number of patients for each diagnosis, sex, age, clinical department, and drug prescriptions. The clinical departments were classified into 12 categories, including 11 departments representing internal medicine and others. Only oral drugs were included in the analysis of prescription patterns, whereas topical and injectable drugs were excluded. Injectable drugs were excluded to avoid the confounding influence of transient acute-phase interventions, such as intravenous fluids, and to prioritize the identification of oral drugs that serve as sustainable markers of nutritional risk in routine clinical practice. Prescribed drugs were classified according to their generic names and the Anatomical Therapeutic Chemical (ATC) classification system. To ensure consistency, the prescription drugs were aggregated using generic names instead of brand names.

### 2.4. Statistical Analyses

Sex differences among patients diagnosed with each condition were assessed using the chi-square (χ^2^) test. Among patients diagnosed with undernutrition or malnutrition, sex differences were further examined across age groups and clinical departments using the chi-square (χ^2^) test. Age was categorized as older adults (≥65 years), younger adults (15–39 years), and Others (0–14 and 40–64 years). The younger adult group was specifically defined based on the Adolescent and Young Adult (AYA) generation classification, a recognized demographic category in Japanese public health. A *p*-value < 0.001 was considered statistically significant. Given the large sample size, *p*-values were expected to be significant in nearly all comparisons; thus, the magnitude of group differences was primarily assessed using the phi coefficient for the effect size. Values of ≥0.1 were considered indicative of meaningful group differences, and larger values were interpreted as reflecting greater between-group differences. Values below this threshold were interpreted as negligible, regardless of statistical significance. Odds ratios (ORs) with 95% confidence intervals (CIs) were calculated to identify drugs that were prescribed significantly more frequently to patients with undernutrition or malnutrition. In this study, crude ORs were calculated for patients who were prescribed at least one oral drug, excluding topical and injectable drugs. Drugs prescribed to <10 patients based on actual patient counts were excluded from the analyses to ensure statistical stability. Data extraction and large-scale aggregations were performed using the AHI partners database’s dedicated online extraction and aggregation system, which is optimized for processing nationwide datasets. All statistical analyses and the generation of descriptive figures were performed using Microsoft Excel (version 16.104; Microsoft Corp., Redmond, WA, USA).

## 3. Results

### 3.1. The Patient Population Diagnosed with Malnutrition and Undernutrition

During the study period from January to December 2024, an estimated 96,673,453 patients were included in the AHI partners database. Among them, 288,112 (0.298%) were diagnosed with undernutrition or malnutrition (Table 1). To ensure that the search settings did not yield patients diagnosed with both malnutrition and undernutrition simultaneously, we adjusted the search criteria to use “or” instead of “and.” None of the patients had protein deficiency disorders or malnutrition-related cataracts. Malnutrition accounted for most cases, whereas undernutrition accounted for a smaller proportion of diagnosed patients. Significant sex differences were observed across all diagnostic categories; however, the effect sizes were minimal.

### 3.2. Sex Differences in Patients with Malnutrition and Undernutrition

**Figure 1:**
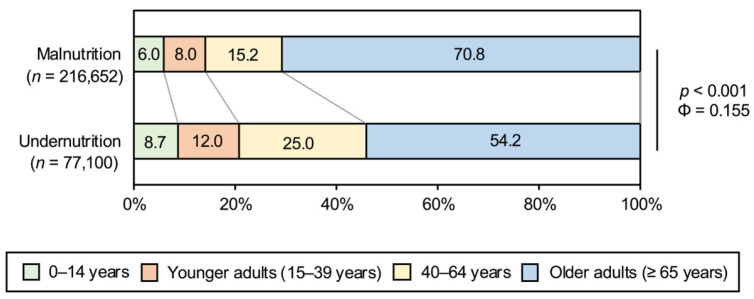
Age distribution of patients with malnutrition and undernutrition. Comparison of age group proportions between the malnutrition (*n* = 216,652) and undernutrition (*n* = 77,100) groups. Age was categorized as older adults (≥65 years), younger adults (15–39 years), and others (0–14 and 40–64 years). Chi-square (χ^2^) tests were used to assess the differences in distribution between the two diagnostic categories, and effect sizes were evaluated using the phi coefficient (Φ).

To examine whether the diagnostic patterns differed across age groups, the age distributions were analyzed within each diagnostic category. Differences in age distribution were observed between the groups (*p* < 0.001, Φ = 0.155), and older adults accounted for more than half of the patients in both diagnostic categories (Figure 1).

To clarify disease-specific sex differences while minimizing the influence of population structure, we analyzed the distribution exclusively within a cohort of patients diagnosed with malnutrition or undernutrition. Unlike the broad comparison against the overall database population in Table 1, this focused approach highlights the diagnostic divergence—the distinct labeling patterns—between the two conditions. Although all comparisons yielded statistically significant differences due to the large sample size, we primarily focused on effect sizes to evaluate the practical magnitude of these differences. Malnutrition was significantly predominant among women (41.2% vs. 58.8%, Φ = 0.236). In contrast, undernutrition was markedly more prevalent among men, with a significant sex difference between the two groups. (70.6% vs. 29.4%, Φ = 0.274). In addition, considering the overall age distribution, analyses were performed to compare older and younger patients to determine whether diagnostic patterns differed between age groups. Among older adults, malnutrition was more prevalent in women than in men (38.3% vs. 61.7%, Φ = 0.0923), whereas undernutrition was more common in men than in women (72.2% vs. 27.8%, Φ = 0.0385). In contrast, among younger adults, women accounted for a higher proportion of malnutrition (33.2% vs. 66.8%, Φ = 0.0481) and undernutrition (46.9% vs. 53.1%, Φ = 0.192). The sex distribution of undernutrition among younger adults showed a reversal in the overall trends. Although undernutrition was more prevalent in men than in women across the entire population, a higher proportion of the malnutrition or undernutrition groups were young women.

### 3.3. Distribution of Clinical Departments

**Figure 2:**
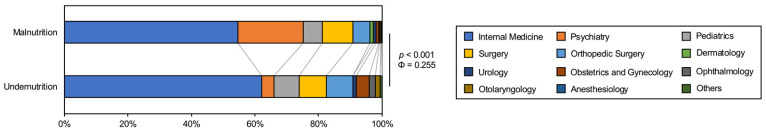
Distribution of clinical departments among patients with malnutrition and undernutrition. Proportion of patients in each clinical department in the malnutrition and undernutrition groups. Chi-square (χ^2^) tests were used to assess diagnostic differences, and effect sizes were evaluated using the phi coefficient (Φ).

A comparison of the clinical department distributions between patients with malnutrition and those with undernutrition revealed a significant difference (*p* < 0.001, Φ = 0.255) (Figure 2). Internal medicine accounted for the largest proportion in both groups. The proportion of patients with psychiatric disorders was substantially higher in the malnutrition group (21.7%) than in the undernutrition group (4.0%). Other departments, including surgery, orthopedics, and urology, showed variable distributions between the two groups.

### 3.4. Drugs Frequently Prescribed to Patients with Malnutrition or Undernutrition

**Figure 3:**
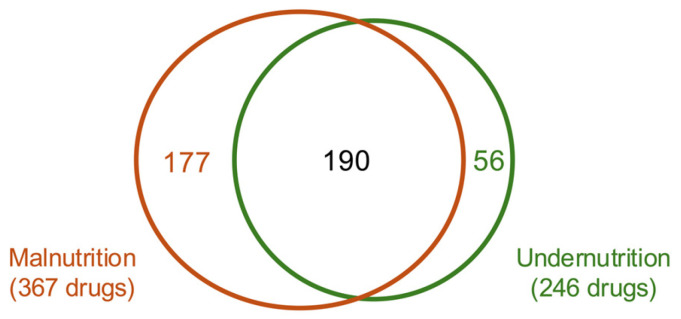
Venn diagram: distribution of drugs prescribed to patients with malnutrition or undernutrition based on generic names (*n* = 423). In total, 367 drugs were prescribed to patients with malnutrition, 246 to patients with undernutrition, and 190 were common to both groups.

To identify patient populations that may be at an increased risk of malnutrition or undernutrition in clinical practice, we examined the drugs that were more frequently prescribed to patients diagnosed with these conditions. Drugs that were prescribed significantly more frequently to patients with malnutrition or undernutrition were identified through prescription analyses (Figure 3). To focus on malnutrition and undernutrition potentially caused by the continuous use of drugs, we examined the related oral drugs, excluding injectable drugs that were expected to be used during hospitalization. Owing to the characteristics of this study, it is necessary to distinguish, based on pharmacological properties, whether the condition was induced by the drug or if the drug was prescribed for treatment. In total, 423 unique agents were identified in patients with malnutrition or undernutrition. Based on their generic names, 367 drugs in the malnutrition group and 246 drugs in the undernutrition group were identified, of which 190 were common to both groups.

Of these, 49 drugs were prescribed to ≥10 patients (actual patient counts). Among the 423 active ingredients prescribed to patients diagnosed with malnutrition or undernutrition, 31 drugs (7.33%) were frequently prescribed, as the lower limit of their OR was >1.00 (Table 2). According to the ATC 1st-level classification, nine categories were observed, with the alimentary tract and metabolism (*n* = 22), nervous system (*n* = 9), and respiratory system (*n* = 5) being the most frequent, although the drugs spanned a wide range of therapeutic classes. Enteral elemental nutrients exhibited the highest OR (OR, 89.7; 95% CI, 88.8–90.6). The enteral elemental formulas consisted of seven products based on the ATC classification code V06B0. These products include various formulations, such as semi-solid and liquid enteral formulas (e.g., ENSURE^®^), and elemental and polymeric formulas, commonly used in clinical practice. Several psychotropic drugs were frequently prescribed, including sulpiride (OR, 19.1) and benzodiazepines, such as lorazepam (OR, 4.31) and diazepam (OR, 3.98). These psychotropic drugs were predominantly prescribed to women (sulpiride, 98%; lorazepam, 96%; and diazepam, 93%). Additionally, gastrointestinal drugs, including sodium ferrous citrate (OR, 15.6), ursodeoxycholic acid (OR, 10.9), proton pump inhibitors, and laxatives, were frequently prescribed.

## 4. Discussion

Undernutrition is often asymptomatic and can be overlooked in clinical settings. Moreover, it has a significant impact on the short-term prognosis. In a nationwide study of hospitalized patients in Japan, undernutrition, as defined by the GLIM criteria, was significantly associated with an increased 30-day mortality risk, with a hazard ratio (HR) of 1.46 (95% CI, 1.33–1.60), and an increased 60-day mortality risk, with an HR of 1.46 (95% CI, 1.34–1.59). Furthermore, approximately half of the patients had incomplete GLIM assessments, and an elevated mortality risk was observed in these patients [19]. Although undernutrition has significant clinical implications, it has not been consistently assessed or managed in clinical settings. In contrast, individualized nutritional support improves clinical outcomes, including survival, suggesting that undernutrition is potentially reversible with appropriate diagnosis and intervention [20].

Although sex differences were observed when undernutrition and malnutrition were analyzed together, considering the large number of patients included in the dataset, the overall sex ratio was nearly equal. However, stratification according to the diagnostic label revealed contrasting sex differences: malnutrition was more common in women than in men, whereas undernutrition was more prevalent in men than in women. These results indicate that the higher prevalence of malnutrition in women observed in this study is consistent with previously reported trends [9,10,11,12]. While physiological factors, such as the influence of sex hormones on appetite regulation [21,22], are often discussed as potential contributors to these nutritional risks in women, our findings primarily reflect how these conditions are recorded in clinical practice. Furthermore, the diagnostic label of “malnutrition” used in this study may not solely reflect undernutrition. Conversely, the higher prevalence of undernutrition in men does not completely align with the findings of previous reports. Recent evidence has shown that the clinical phenotypes of undernutrition differ according to sex, and individuals without evident weight loss or BMI reduction may still be diagnosed as undernourished [23].

Therefore, these results likely reflect sex-specific differences in diagnostic and recording practices, rather than true prevalence differences. The observed sex differences by diagnostic label suggest that malnutrition and undernutrition are not single clinical entities but rather complex states comprising diverse etiologies and phenotypes. International discussions on adult malnutrition have noted that ICD classifications do not fully capture the current clinical reality of undernutrition, particularly among older adults. Although the transition to ICD-11 is globally underway, the current edition lacks comprehensive diagnostic codes that align with the modern clinical definitions of malnutrition in adults. Currently, the primary concept of ICD-11 for adult malnutrition remains heavily focused on low BMI and fails to account for patients with malnutrition with normal or high BMI. In response to these limitations, over 40 clinical nutrition societies worldwide, in collaboration with the Swedish National Board of Health and Welfare, submitted a proposal to the WHO in late 2020 to amend ICD-11 [24]. Consequently, in October 2025, the WHO officially decided that, starting in 2027, the ICD-11 will incorporate a GLIM-inspired code (5B72) for the diagnosis of “Undernutrition in Adults.” Our findings provide timely real-world data to support this international shift toward more standardized and comprehensive diagnostic frameworks. Globally, westernized diets have led to obesity concerns; however, in Japan, the “underweight” status of young women is recognized as a public health concern. According to the 2023 National Health and Nutrition Survey, 12.2% of women had a BMI ≤ 18.5 kg/m^2^, with 20–30-year-old women showing a prevalence of 20.2% [7]. The contributing factors include an unbalanced and excessive diet. Undernutrition in young adulthood may affect health, including bone formation [25]. Additionally, undernutrition is associated with psychiatric symptoms, and women are at a higher risk of depression, which may contribute to eating disorders and impaired nutritional status. Therefore, the Japan Society for the Study of Obesity released a statement on the Female Underweight/Undernutrition Syndrome to maintain health throughout a woman’s life [26]. In the current study, a potential overlap with psychiatric comorbidities was supported among patients with malnutrition, although the causality would require adjustment for comorbidities and drugs.

To characterize the clinical background of patients diagnosed with malnutrition or undernutrition, we compared the prescription patterns of patients without these diagnoses. 31 drugs were prescribed significantly more frequently to patients with malnutrition or undernutrition. As expected, the highest ORs were observed for enteral elemental formulas in patients requiring nutritional support. These drugs are used for postoperative nutritional management or when oral intake becomes difficult. Similarly, our previous analysis using a database of near-miss events from community pharmacies revealed higher prescription rates of enteral elemental formulas among patients requiring enteral nutrition [27]. This database analysis likely reflects actual clinical prescription patterns. Other significant drugs span multiple therapeutic categories. Antipsychotics, such as sulpiride, lorazepam, and diazepam, which are frequently prescribed for psychiatric symptom management, anxiety, and sleep disorders, were more common in these patients. Our analysis of the prescribing departments revealed that sulpiride and lorazepam were most frequently prescribed by the psychiatric department. Although sulpiride is indicated for the treatment of both peptic ulcers and psychiatric conditions, the most prescriptions in this cohort originated from psychiatric departments. Regarding the co-occurrence of these conditions, previous studies have established an association between malnutrition and depression [28,29,30]. In clinical settings, certain drugs may be related to nutritional status by potentially affecting appetite or altering gastrointestinal motility; however, their impact is often underestimated. Our previous study using a spontaneous reporting database revealed that various therapeutic categories were significantly associated with decreased appetite and taste disorders [31]. Therefore, a comprehensive review of drug profile is essential for effective nutritional management. The prescription patterns observed in this study further support the clinical profiles suggested by the demographic analyses, highlighting the potential comorbidities and interventions in patients with undernutrition.

This study had some limitations. First, the database lacks specific clinical parameters, such as BMI or laboratory values, which are essential for the direct assessment of nutritional status. Although sex- and age-stratified analyses were performed, other potential confounders, including comorbidities, lifestyle factors, and socioeconomic status, were not fully adjusted. Second, as this was an observational study, we could not directly establish a causal association between malnutrition and drug prescription. Furthermore, the drug findings were based on crude odds ratios and were not adjusted for other variables, which may limit the ability to draw definitive conclusions regarding specific drug effects. The prescription data cannot distinguish whether drugs were administered for therapeutic purposes or whether they contributed to undernutrition as an adverse effect. Moreover, the prescription information does not necessarily reflect actual drug intake or adherence. Third, the patient estimation database used in this study was based on extrapolated data, with nationwide estimated values calculated using correction coefficients that reflected factors such as prefecture and age. Therefore, these correction factors may disproportionately affect patients with rare diseases. Finally, diagnostic codes for “malnutrition” or “undernutrition” often have low priority in clinical documentation, which may result in an underestimation of the actual number of affected patients. Our findings should be interpreted as indicative of trends in diagnostic labeling behaviors and prescription patterns among patients with undernutrition rather than definitive causal associations.

## 5. Conclusions

This study is the first nationwide analysis in Japan to examine diagnostic labeling patterns, sex differences, and drug prescription trends among patients with malnutrition or undernutrition using a patient estimation database. Our findings revealed significant sex-specific differences: undernutrition predominated in men, whereas malnutrition was more prevalent in women. These results suggest that diagnostic labels may reflect clinical labeling practices rather than purely physiological differences. The inconsistency in diagnostic criteria underscores the potential importance of standardized frameworks, such as the GLIM criteria, for achieving more consistent clinical assessments. Furthermore, integrating sex-specific perspectives into diagnostic considerations may further support individualized care and help optimize patient outcomes across the life course.

## Figures and Tables

**Table 1:** Number of patients diagnosed with malnutrition and undernutrition.

Diagnostic Category	Total (%)	Men (%)	Women (%)	*p*-Value	Φ
Undernutrition or Malnutrition	288,112 (100)	138,140 (47.9)	149,972 (52.1)	<0.001	0.013
Malnutrition	216,652 (100)	89,200 (41.2)	127,452 (58.8)	<0.001	0.018
Undernutrition	77,100 (100)	54,423 (70.6)	22,677 (29.4)	<0.001	0.006
All registered patients	96,673,453 (100)	57,688,431 (59.7)	38,985,022 (40.3)		

Chi-square (χ^2^) tests were used to assess sex differences, with expected counts derived from the estimated overall sex distribution (men/women) in the database population. Effect sizes were evaluated using the phi coefficient (Φ).

**Table 2:** Drugs were prescribed significantly more frequently to patients with malnutrition or undernutrition.

ATC Classification	Generic Name	Total Patients	Men (%)	Women (%)	Odds Ratio [95% CI]
V06B0	Enteral elemental formula	62,526	69	31	89.7 [88.8–90.6]
A02B9	Sulpiride	30,731	1.7	98	19.1 [18.8–19.3]
B03A1	Sodium ferrous citrate	40,044	5.0	95	15.6 [15.4–15.7]
A05A2	Ursodeoxycholic acid	30,920	93	7.2	10.9 [10.7–11.0]
N05B1	Triclofos sodium	2272	9.2	91	9.59 [9.20–10.0]
A02B2	Vonoprazan fumarate	66,036	36	64	7.65 [7.59–7.72]
A03F0	Mosapride citrate hydrate	36,942	5.0	95	7.52 [7.44–7.61]
N03A0	Levetiracetam	5940	80	21	6.75 [6.58–6.93]
A02B9	Polaprezinc	7483	56	45	6.46 [6.31–6.61]
N05B1	Lemborexant	38,667	77	23	6.35 [6.28–6.42]
A07H0	Loperamide hydrochloride	12,176	91	9.0	5.67 [5.57–5.77]
A07E1	Mesalazine	5306	82	18	5.04 [4.91–5.18]
A07F0	Butyric acid bacteria	69,420	32	68	4.63 [4.59–4.67]
N05C0	Lorazepam	6942	4.3	96	4.31 [4.21–4.42]
H03A0	Levothyroxine sodium hydrate	12,883	37	64	4.04 [3.97–4.12]
N05C0	Diazepam	4687	6.7	93	3.98 [3.87–4.10]
A02B2	Lansoprazole	29,192	66	34	3.92 [3.88–3.97]
N01B3	Lidocaine hydrochloride	25,521	84	16	3.83 [3.78–3.88]
N05A1	Risperidone	5694	72	28	3.25 [3.17–3.34]
A11C2	Alfacalcidol	7168	40	60	3.07 [3.00–3.15]
N03A0	Sodium valproate	4415	14	86	2.35 [2.28–2.42]
C10A1	Rosuvastatin calcium	40,878	26	74	2.34 [2.31–2.36]
A06A7	Macrogol 4000, sodium chloride, sodium bicarbonate, potassium chloride	3675	54	46	2.32 [2.25–2.40]
A02A2	Dimeticone	31,836	83	17	2.15 [2.12–2.17]
A02B2	Esomeprazole	17,705	65	35	2.00 [1.97–2.03]
A07F0	Lactomin, butyric acid bacteria, amylolytic bacillus	12,695	92	7.8	1.88 [1.85–1.92]
V03B1	Rikkunshito	2727	61	39	1.85 [1.78–1.92]
C08A0	Amlodipine	40,142	15	86	1.51 [1.50–1.53]
A02A1	Sodium bicarbonate	10,874	70	30	1.29 [1.26–1.31]
A06A2	Sodium picosulfate hydrate	11,321	68	32	1.25 [1.23–1.28]
V03H0	Pronase	10,958	70	30	1.20 [1.18–1.23]

The 31 drugs prescribed to patients with malnutrition or undernutrition were ranked in descending order of odds ratio (OR). Drugs prescribed to <10 patients (actual patient counts) were excluded from the analysis to avoid reduced reliability of the results due to small sample sizes.

## Data Availability

The data analyzed in this study were proprietary and used under a license from AHI Partners Inc. Therefore, the data are not publicly available owing to commercial confidentiality and legal restrictions.

## Abbreviations

The following abbreviations are used in this manuscript.

ATC	Anatomical Therapeutic Chemical
AYA	Adolescent and Young Adult
BMI	Body Mass Index
CI	Confidence Interval
GLIM	Global Leadership Initiative on Malnutrition
HR	Hazard Ratio
ICD-10	International Statistical Classification of Diseases and Related Health Problems, 10th Revision
MHLW	Ministry of Health, Labour and Welfare
OR	Odds Ratio
WHO	World Health Organization
